# Routine-recorded physical activity and screening-defined cardio-renal-metabolic vulnerability amongst older adults in a cold-climate agricultural county in Northeast China

**DOI:** 10.3389/fpubh.2026.1831345

**Published:** 2026-07-07

**Authors:** Zhuangzhuang Guo, Yaru Zhong, Yongheng Zhao, Xuefeng Xi, Gaixia Hou, Limeng Liu, Dehui Zhang

**Affiliations:** 1 School of Wushu, Henan University, Kaifeng, China; 2 Graduate School, Kyungil University, Gyeongsan-si, Republic of Korea; 3 School of Physical Education and Health Science, Mudanjiang Normal University, Mudanjiang, China; 4 Dongcheng Community Health Service Center, Wangkui County, Heilongjiang, China

**Keywords:** cardio-renal-metabolic vulnerability, cold-climate agriculture, health examination, older adults, routine-recorded physical activity, rural health, screening-defined outcome

## Abstract

**Background:**

Older adults in rural settings often show overlapping cardiovascular, renal, and metabolic abnormalities, yet the meaning of routine-recorded physical activity in health-examination data remains uncertain.

**Objective:**

To examine the association between routine-recorded physical activity and screening-defined cardio-renal-metabolic (CRM) vulnerability amongst older adults in Wangkui County, China.

**Methods:**

This community-based cross-sectional study used data from the 2025 annual health examination programme for adults aged ≥65 years. Physical activity was derived from a routine health-examination item and treated as a coarse activity-status indicator, not as a validated measure of exercise dose, intensity, energy expenditure, or activity domain. The primary exposure was inactive versus active; the three-category classification was retained for secondary analysis. The primary outcome was high screening-defined CRM vulnerability, defined as involvement of at least two cardiovascular, metabolic, and broad renal–urinary domains; this was an operational screening construct, not a clinically validated disease phenotype. Modified Poisson regression with robust standard errors estimated prevalence ratios (PRs), with multiple imputation for primary analyses.

**Results:**

Amongst participants with non-missing activity data, 852 were inactive and 1,363 were active. In the fully adjusted multiply imputed model, active routine-recorded activity was associated with higher prevalence of high CRM vulnerability than inactivity (PR = 1.121, 95% CI: 1.051–1.197). Similar patterns were observed for severe and subclinical high CRM vulnerability. Domain-specific analyses showed that the positive pattern was observed mainly for cardiovascular burden and broad renal–urinary screening signals, whereas metabolic and stricter renal indicators were weaker or less consistent.

**Conclusion:**

Being recorded as active in the routine health-examination activity item co-occurred with greater screening-defined CRM vulnerability. This finding should be interpreted as a context-sensitive screening association, not evidence that physical activity causes CRM vulnerability or that structured physical activity is harmful. The renal–urinary signal should be interpreted conservatively because UACR was unavailable and the broader renal–urinary pattern was observed primarily in relation to urine occult blood positivity.

## 1 Introduction

Population ageing has shifted the public health burden in older adults from isolated diseases towards the co-occurrence of multi-system abnormalities and cumulative vulnerability (1, 2). In later life, cardiovascular, renal, and metabolic disturbances frequently cluster and interact, generating broader risk profiles that may not be adequately captured by single-disease indicators alone (3, 4). This perspective is particularly relevant in county-level screening systems, especially in rural settings, where older adults often present with overlapping abnormalities rather than neatly separated diagnostic categories (5). Accordingly, a screening-defined cardio-renal-metabolic (CRM) vulnerability framework may provide a more informative public health lens for identifying older adults who may warrant further screening review or follow-up prioritisation than reliance on any one condition in isolation (6).

Physical activity is widely recognised as an important component of healthy ageing and chronic disease prevention (7, 8). However, the health meaning of activity in later life depends on its social and functional context (9, 10). Much of the existing evidence linking higher physical activity to better health outcomes has been derived from leisure-time, structured, or health-promoting activity, whereas less is known about the public health meaning of physical activity when it is captured through coarse routine health-examination records in rural older populations (10, 11). In rural older populations, routine-recorded physical activity may encompass heterogeneous daily movement, including domestic tasks, agricultural routines, transport-related activity, and livelihood-related demands, without distinguishing these domains from discretionary exercise (12). Under such conditions, an “active” routine record should not automatically be interpreted as structured health-promoting exercise; it may also coexist with cumulative workload, constrained recovery, and limited access to health-promoting resources (13, 14).

These issues are especially salient in cold-climate agricultural settings such as Wangkui County, Heilongjiang Province, in Northeast China. In such environments, ageing, daily activity context, and health remain tightly intertwined (15). Older adults may appear active in routine records because of continuing household responsibilities, agricultural routines, transport-related movement, livelihood demands, or leisure-time activity, but the routine record itself does not distinguish these sources (16). At the same time, county-level annual health examination programmes offer a valuable real-world platform for capturing multi-system screening profiles in community-dwelling older adults (17). Yet evidence remains limited on whether being recorded as active in a routine health-examination activity item corresponds to a more favourable or less favourable screening-defined cardio-renal-metabolic profile (18). In particular, few studies have examined this question using a screening-defined composite vulnerability framework, and even fewer have described which domain-specific screening patterns accompany any observed association (19, 20).

Therefore, the present study examined the association between routine-recorded physical activity status and high screening-defined CRM vulnerability amongst older adults in Wangkui County, Heilongjiang Province. Because the activity item was derived from routine health-examination records and did not distinguish occupational, agricultural, domestic, transport-related, or leisure-time physical activity, the measured exposure was interpreted as a coarse routine-recorded activity indicator rather than as a domain-specific physical activity measure. We further evaluated whether the observed association was robust across alternative vulnerability definitions and whether it corresponded to specific domain-level screening patterns rather than reflecting a generalised multisystem screening pattern. By doing so, this study aimed to provide context-sensitive evidence for interpreting routine-recorded physical activity at the intersections of ageing, activity context, and health in a cold-climate rural population (21).

Building on but distinct from our previous publication using the same county-level routine health-examination platform, the present study addresses a different research question (22). The previous article focused on prevalent hypertension, blood-pressure-free obesity–metabolic phenotypes, a cohort-specific physical activity volume index, and leakage-free machine-learning models for hypertension prediction. In contrast, the present study shifts the analytic focus from a single disease outcome to a screening-defined multidomain cardio-renal-metabolic vulnerability construct. Specifically, we examined whether routine-recorded physical activity status was associated with the co-occurrence of cardiovascular, metabolic, and renal–urinary screening burdens, and whether this association corresponded to specific domain-level screening patterns rather than reflecting hypertension-related information alone.

## 2 Methods

### 2.1 Study design and population

This community-based cross-sectional study was conducted in Wangkui County, Heilongjiang Province, China, and is reported in accordance with the Strengthening the Reporting of Observational Studies in Epidemiology (STROBE) guidelines for observational research (23). Data were derived from the 2025 government-organised annual health examination programme for community-dwelling older adults. This programme provides free routine health examinations for residents aged 65 years and older and includes standardised questionnaires, physical examinations, laboratory testing, and chronic disease screening conducted at township health centres or village clinics.

All examinations were performed by uniformly trained physicians and nurses according to national technical specifications for basic public health services and chronic disease surveillance (23). Blood samples were analysed in a county-level central clinical laboratory operating under internal and external quality-control procedures. The anonymised dataset was released to the research team under a data-use agreement with the local Health Bureau. The study complied with the Declaration of Helsinki and was approved by the Biomedical Research Ethics Subcommittee of Henan University (approval no. HUSOM2025-929) and the local Health Bureau. All participants provided written informed consent at the time of the health examination.

**Figure 1:**
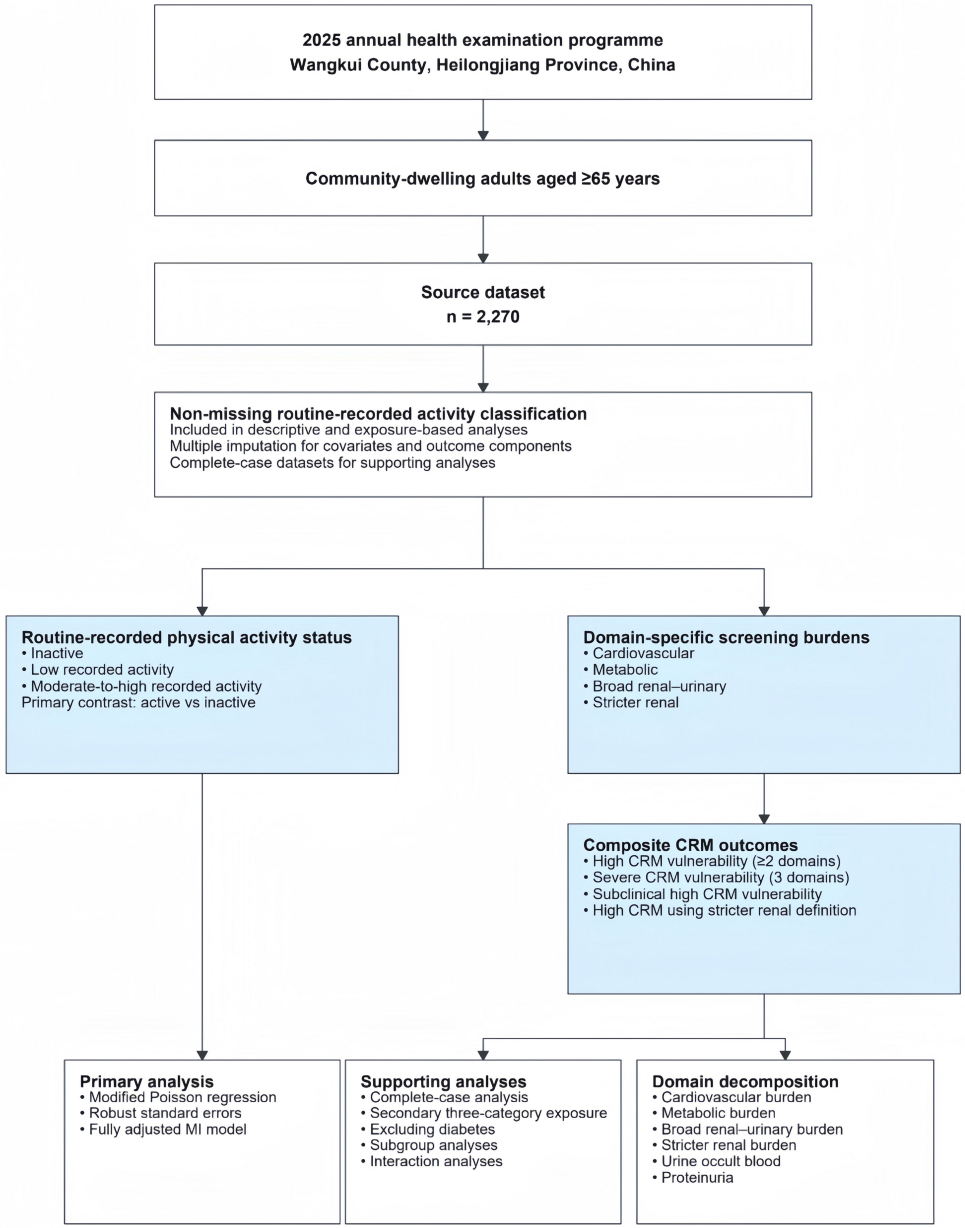
Study flow and analytic framework for routine-recorded physical activity and screening-defined cardio-renal-metabolic vulnerability in older adults from Wangkui County.

A total of 2,270 older adults were included in the source dataset. Unlike prior disease-specific analyses based on this county-level screening platform, the present study was designed to examine screening-defined cardio-renal-metabolic (CRM) vulnerability as a multisystem composite construct and to evaluate its association with routine-recorded physical activity status in this ageing rural population. Participants with non-missing routine-recorded physical activity classification were included in exposure-based descriptive and regression analyses. Missing covariates and outcome components were handled using multiple imputation for the primary inferential analyses, whereas the activity exposure itself was not imputed. Complete-case analyses were additionally conducted as supporting analyses. The overall study flow, variable construction, and analytic framework are summarised in Figure 1.

### 2.2 Relationship with previous publication

The present analysis was conducted using the same 2025 government-organised annual health-examination source platform in Wangkui County, Heilongjiang Province, China, as our previous Frontiers in Public Health article titled “Labour-Type Physical Activity, Metabolic Dysregulation, and Hypertension in Rural Older Adults: Rethinking Work, Exercise, and Health in a Cold-Climate Agricultural Community (22).” The same source population of 2,270 older adults was used as the underlying screening platform. However, the analytic aims, exposure operationalization, outcome hierarchy, and statistical approach differed from those of the previous study.

The previous article used a complete-case analytic sample of 2,191 participants and focused on prevalent hypertension, blood-pressure-free obesity–metabolic phenotypes, a cohort-specific physical activity volume index based on frequency and duration, and leakage-free machine-learning models for hypertension prediction. The current study used the same source platform but focused on screening-defined cardio-renal-metabolic vulnerability as a multidomain composite construct. Participants with non-missing routine-recorded activity classification were included in exposure-based analyses, and missing covariates and outcome components were addressed using multiple imputation. The primary exposure was a coarse inactive-versus-active routine-recorded activity indicator rather than a quantitative physical activity volume index. The primary outcome was high screening-defined cardio-renal-metabolic vulnerability, defined as involvement of at least two prespecified cardiovascular, metabolic, and renal–urinary screening domains.

Therefore, the current manuscript was not designed to duplicate the previous hypertension-focused analysis. Instead, it evaluates whether a routine activity record has broader screening meaning for multidomain cardio-renal-metabolic vulnerability in older adults and clarifies the domain-level contributors to this composite vulnerability construct.

### 2.3 Variables and measurements

#### 2.3.1 Demographic and lifestyle variables

Age (years) and sex (male/female) were obtained from registration records and verified during face-to-face interviews by trained staff. Age was analysed as a continuous variable. Sex was coded as a binary indicator. Smoking and drinking variables were extracted from the health-examination questionnaire and harmonised into current versus non-current categories for multivariable analyses.

#### 2.3.2 Anthropometric and biochemical measurements

Body weight and height were measured using calibrated scales and stadiometers with participants wearing light clothing and no shoes. Body mass index (BMI) was calculated as weight in kilogrammes divided by height in metres squared. For quality control, implausible BMI values were excluded using a prespecified physiologic range of 14–60 kg/m^2^.

After an overnight fast, venous blood samples were collected and processed according to routine county-level laboratory procedures. Fasting plasma glucose (FPG), triglycerides (TG), and high-density lipoprotein cholesterol (HDL-C) were measured using standard enzymatic methods on automated analysers following the manufacturer’s protocols and local quality-control standards (23). Recorded estimated glomerular filtration rate (eGFR) values were extracted directly from the health-examination records. Urine protein and urine occult blood were recorded as binary screening indicators.

#### 2.3.3 Blood pressure and chronic disease records

Blood pressure was measured using calibrated automated sphygmomanometers after seated rest in accordance with routine screening procedures. Systolic blood pressure (SBP) and diastolic blood pressure (DBP) were extracted from the examination records. Information on hypertension history, diabetes history, antihypertensive treatment, glucose-lowering treatment, and lipid-lowering treatment was also extracted from the health-examination forms and harmonised to binary variables.

#### 2.3.4 Routine-recorded physical activity

Routine-recorded physical activity status was derived from the lifestyle section of the county health-examination form. The source field was translated as “frequency of physical exercise.” The same section also included companion exercise-related fields on exercise duration per session, years of continued exercise, and exercise type. In the exported analytic dataset, the observed non-missing raw values for the physical-exercise frequency field were 0, 1, and 3; no raw value of 2 was observed. Participants with missing frequency records were treated as having missing exposure status and were not included in exposure-based regression models.

The analytic recoding was based on the exported frequency values and was further checked against the companion exercise-related fields. Raw value 0 was classified as inactive because none of the participants with this value had recorded exercise duration, years of continued exercise, or exercise type. Raw values 1 and 3 were classified as active because most participants with these values had non-missing companion exercise records. For the original three-category exposure, raw value 0 was classified as inactive, raw value 1 as low recorded activity, and raw value 3 as moderate-to-high recorded activity. The source field, translated item label, exported raw values, companion-field evidence, analytic recoding rules, and sample sizes are reported in Supplementary Tables S1 and S2.

Because this item was collected as part of a routine public health screening programme rather than through a validated physical activity questionnaire, it was treated as a coarse routine-recorded physical activity indicator. It was not interpreted as a quantitative measure of total physical activity, exercise dose, energy expenditure, intensity, duration, or a specific activity domain. Although companion fields on exercise duration, years of continued exercise, and exercise type were available, these fields did not provide a validated domain-specific physical activity assessment and were not used to construct the primary exposure. The routine record did not distinguish leisure-time exercise, occupational or agricultural activity, domestic work, transport-related activity, or livelihood-related movement. Therefore, “routine-recorded physical activity” is used throughout the manuscript to describe the measured exposure, whereas labour- or work-related interpretations are restricted to the contextual discussion of the rural agricultural setting.

For the primary analysis, the exposure was defined as inactive versus active. Participants with raw value 0 were classified as inactive, whereas participants with raw values 1 and 3 were combined as active. This binary contrast was selected because the low recorded activity subgroup was small in the observed cohort. The original three-category classification — inactive, low recorded activity, and moderate-to-high recorded activity — was retained only as a secondary sensitivity analysis to examine whether the direction of association was consistent across the original recorded activity levels. Throughout this manuscript, routine-recorded physical activity refers only to the exported health-examination activity-frequency record. It should not be interpreted as a validated physical activity questionnaire, exercise-dose measure, total energy-expenditure estimate, intensity measure, or domain-specific activity assessment.

#### 2.3.5 Domain-specific screening burdens

Three domain-specific screening burdens were defined *a priori*.

Cardiovascular burden was defined as the presence of any of the following: SBP ≥ 140 mmHg, DBP ≥ 90 mmHg, hypertension history, or antihypertensive treatment.

Metabolic burden was defined as the presence of any of the following: FPG ≥ 7.0 mmol/L, diabetes history, glucose-lowering treatment, TG ≥ 1.7 mmol/L, low HDL-C, or lipid-lowering treatment. Low HDL-C was defined using sex-specific thresholds of <1.0 mmol/L in men and <1.3 mmol/L in women.

Broad renal–urinary screening burden was defined as reduced recorded eGFR (<60 mL/min/1.73 m^2^), proteinuria positivity, or urine occult blood positivity. UACR was not available in the routine health-examination records. Therefore, the renal–urinary domain should be interpreted as a broad screening signal rather than as an albuminuria-based or clinically adjudicated kidney disease definition.

A stricter renal screening burden variable was also defined for sensitivity analyses using only reduced recorded eGFR (<60 mL/min/1.73 m^2^) and/or proteinuria positivity. This variable was interpreted as a screening-level renal signal rather than as clinically adjudicated chronic kidney disease.

#### 2.3.6 Composite cardio-renal-metabolic vulnerability outcomes

The primary outcome was high screening-defined cardio-renal-metabolic (CRM) vulnerability, defined as involvement of at least two of three prespecified domains: cardiovascular burden, metabolic burden, and broad renal–urinary screening burden. This composite framework was intended to capture multisystem screening-level vulnerability within a county-based examination context rather than a clinically adjudicated cardio-renal-metabolic syndrome.

A secondary composite outcome, severe CRM vulnerability, was defined as concurrent involvement of all three domains. A further alternative outcome, subclinical high CRM vulnerability, was defined as involvement of at least two domains using objective screening abnormalities only, without relying on prior disease history or treatment status. For sensitivity analyses, high CRM vulnerability using a stricter renal definition was constructed by replacing the broad renal–urinary screening domain with the strict renal burden domain. Because the broad renal–urinary domain included urine occult blood positivity in addition to reduced eGFR and proteinuria, the composite outcome should be interpreted as a screening-defined multisystem vulnerability construct rather than as evidence of established cardio-renal disease burden. The operational definitions of the exposure, domain-specific screening burdens, and composite CRM vulnerability outcomes are summarised in Supplementary Table S3. These composite CRM outcomes were designed to describe screening-level co-occurrence of routine examination abnormalities. They were not intended to diagnose cardio-renal-metabolic disease, define a clinically validated disease phenotype, or establish prognosis.

### 2.4 Data preparation and quality control

All analyses were performed in R (version 4.5.0; R Foundation for Statistical Computing, Vienna, Austria). Data management and variable harmonisation followed a prespecified analytic workflow. The raw de-identified dataset was imported and cleaned using standardised scripts. Continuous variables were checked for physiologic plausibility, and implausible values were set to missing according to prespecified limits. Binary variables were harmonised to 0/1 coding, and categorical exposure variables were recoded with fixed reference levels. For activity exposure, the binary inactive-versus-active variable was constructed as the primary analytic exposure, and the original three-category variable was retained for secondary analyses. The activity exposure itself was not imputed.

A primary analysis dataset was constructed by retaining variables required for the prespecified exposure, outcome, and covariate models. Because missingness was present in several covariates and outcome components, complete-case and multiply imputed analytic datasets were both prepared according to the prespecified workflow. The routine-recorded physical activity grouping itself was not imputed. Instead, relevant component variables were imputed and the composite vulnerability outcomes were reconstructed after imputation. Detailed handling of missing data is described in Section 2.5.6.

### 2.5 Statistical analysis

#### 2.5.1 Descriptive statistics

Participant characteristics were summarised primarily according to binary routine-recorded physical activity status: inactive versus active. Continuous variables were described using means, and categorical variables were described using percentages. The original three-category activity distribution was additionally summarised in the Supplementary material because the low-activity subgroup was small. The distribution of the primary and secondary vulnerability outcomes across binary activity status was examined descriptively.

#### 2.5.2 Primary regression analysis

The primary analysis examined the association between binary routine-recorded physical activity status and high screening-defined CRM vulnerability. The exposure contrast was active versus inactive, with inactive participants as the reference group. Because the study was cross-sectional and the outcomes were prevalent rather than incident, the analyses were designed to estimate prevalence ratios and screening-defined co-occurrence patterns rather than causal effects. Poisson regression models with robust standard errors were used to estimate prevalence ratios (PRs) and 95% confidence intervals (CIs), providing directly interpretable association measures for common outcomes in a cross-sectional setting (24).

Three hierarchical models were fitted: an unadjusted model; a model adjusted for age, sex, smoking, and drinking; and a fully adjusted model additionally including BMI. BMI was included as a pragmatic adiposity-related covariate in the main adjustment set, whilst recognising that its role in the analytic framework may be complex in this setting. Accordingly, adjusted estimates were interpreted as association measures rather than direct effect estimates. The fully adjusted multiply imputed binary-exposure model was treated as the primary inferential model.

#### 2.5.3 Sensitivity analyses

Several sensitivity analyses were prespecified to assess the robustness of the primary association. First, the primary binary-exposure modelling strategy was repeated for severe CRM vulnerability, subclinical high CRM vulnerability, and high CRM vulnerability using the stricter renal definition. Second, the original three-category activity variable was examined as a secondary sensitivity analysis, with inactive participants as the reference group, to preserve information from the routine record whilst recognising that the low-activity subgroup was small and estimates for this category were expected to be imprecise. Third, analyses excluding participants with diabetes were performed. Fourth, complete-case models were used as supporting analyses to compare the direction and magnitude of estimates with the multiply imputed results.

#### 2.5.4 Domain-specific analyses

To describe which domain-specific indicators aligned with the observed composite association and to distinguish selective from generalised multidomain screening patterns, separate robust Poisson models were fitted for cardiovascular burden, metabolic burden, broad renal–urinary screening burden, strict renal burden, eGFR <60 mL/min/1.73 m^2^, urine occult blood positivity, and proteinuria positivity. These models used binary routine-recorded physical activity status as the exposure and followed the same covariate structure as the primary fully adjusted model.

#### 2.5.5 Subgroup and interaction analyses

Subgroup analyses for the primary outcome were conducted by sex, age group, and BMI group. Age was stratified as <75 versus ≥75 years, and BMI was stratified using the prespecified supporting-analysis cut-point. Within each stratum, fully adjusted multiply imputed models using binary routine-recorded physical activity status were fitted. Formal interaction analyses were additionally conducted by including interaction terms between binary routine-recorded physical activity status and sex, age group, or BMI group in the corresponding regression models. These subgroup and interaction analyses were considered exploratory and were interpreted cautiously.

#### 2.5.6 Missing data

Missing covariates and outcome components were addressed using multiple imputation with chained equations (25). Twenty imputed datasets were generated, and imputation models were specified according to variable type. The routine-recorded physical activity exposure itself was not imputed. Instead, participants with missing activity classification were not included in exposure-based regression models, whereas relevant covariates and outcome component variables were imputed and the composite CRM vulnerability outcomes were recalculated within each imputed dataset after imputation. Estimates were pooled across imputed datasets for the primary binary-exposure analyses. Complete-case analyses were performed as supporting comparisons to assess consistency in direction and magnitude.

## 3 Results

### 3.1 Participant characteristics

Participants were summarised according to the revised primary exposure contrast: inactive versus active routine-recorded physical activity. Baseline characteristics are presented in Table 1. The active group combined participants with low and moderate-to-high routine-recorded physical activity, whereas participants with missing physical-activity classification were excluded from this descriptive table. These comparisons were intended to characterise the analytic sample rather than to support formal between-group hypothesis testing.

**Table 1:** Baseline characteristics of participants according to the revised binary routine-recorded physical activity classification.

Characteristic	Inactive	Active
*n*	852	1,363
Age, years	74.52	71.58
Male, %	49.30	48.49
BMI, kg/m^2^	23.74	25.01
SBP, mmHg	142.53	147.60
DBP, mmHg	84.58	88.07
FPG, mmol/L	6.00	6.25
TG, mmol/L	1.90	1.86
HDL-C, mmol/L	1.36	1.38
eGFR, mL/min/1.73 m^2^	73.43	77.02
Current smoking, %	8.80	13.58
Current drinking, %	1.89	1.85
Urine occult blood positive, %	26.64	34.19
Proteinuria positive, %	4.34	2.49
High CRM vulnerability, %	60.09	70.14
Severe CRM vulnerability, %	18.66	24.43
Subclinical high CRM vulnerability, %	52.26	60.63

Values are presented as means for continuous variables and percentages for categorical variables. These descriptive comparisons were not used for formal between-group hypothesis testing. The revised primary exposure was binary routine-recorded physical activity status. Participants with low and moderate-to-high routine-recorded physical activity were combined into the active group, and inactive participants were used as the reference category in subsequent regression analyses. Participants with missing physical activity classification were excluded from this descriptive table. High cardio-renal-metabolic vulnerability was defined as involvement of at least two of the cardiovascular, metabolic, and broad renal–urinary domains; severe CRM vulnerability as involvement of all three domains; and subclinical high CRM vulnerability as involvement of at least two domains based on objective screening abnormalities only. BMI, body mass index; SBP, systolic blood pressure; DBP, diastolic blood pressure; FPG, fasting plasma glucose; TG, triglycerides; HDL-C, high-density lipoprotein cholesterol; eGFR, estimated glomerular filtration rate; CRM, cardio-renal-metabolic.

Compared with inactive participants, active participants were generally younger and had higher mean BMI and systolic blood pressure. They also showed a higher descriptive prevalence of urine occult blood positivity, high screening-defined cardio-renal-metabolic (CRM) vulnerability, severe CRM vulnerability, and subclinical high CRM vulnerability, whereas proteinuria positivity was less frequent. Mean fasting plasma glucose and triglyceride levels were broadly similar between groups. The original three-category activity distribution, including the small low recorded-activity subgroup, is documented in Supplementary Tables S1 and S2 and retained only for secondary sensitivity analyses.

### 3.2 Routine-recorded physical activity and screening-defined cardio-renal-metabolic vulnerability

**Figure 2:**
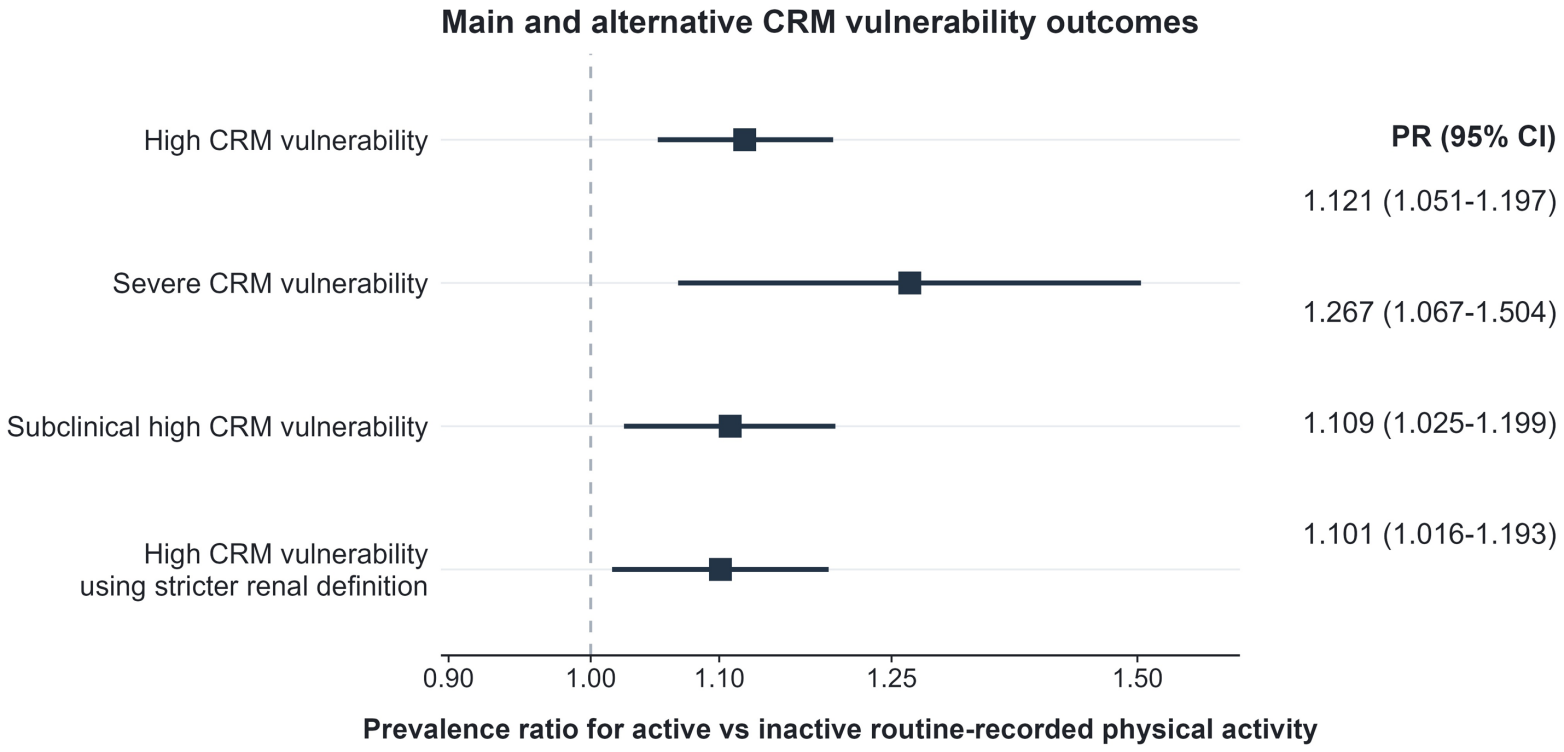
Associations of routine-recorded physical activity with main and alternative screening-defined cardio-renal-metabolic vulnerability outcomes. Forest plot showing prevalence ratios and 95% confidence intervals for the associations of active versus inactive routine-recorded physical activity with high screening-defined cardio-renal-metabolic vulnerability, severe cardio-renal-metabolic vulnerability, subclinical high cardio-renal-metabolic vulnerability, and high cardio-renal-metabolic vulnerability using the stricter renal definition. Estimates are from fully adjusted multiply imputed modified Poisson regression models with robust standard errors. Models were adjusted for age, sex, current smoking, current drinking, and body mass index. Inactive participants were used as the reference group. CRM, cardio-renal-metabolic; PR, prevalence ratio; CI, confidence interval.

Associations between binary routine-recorded physical activity and screening-defined CRM vulnerability are presented in Table 2 and Figure 2. In the fully adjusted multiply imputed model, active routine-recorded physical activity was associated with a higher prevalence of high CRM vulnerability compared with inactivity (PR = 1.121, 95% CI: 1.051–1.197). Similar positive directions of association were observed for severe CRM vulnerability (PR = 1.267, 95% CI: 1.067–1.504), subclinical high CRM vulnerability (PR = 1.109, 95% CI: 1.025–1.199), and high CRM vulnerability using the stricter renal definition (PR = 1.101, 95% CI: 1.016–1.193). The full hierarchical binary models are provided in Supplementary Table S4.

**Table 2:** Primary binary associations between routine-recorded physical activity and screening-defined cardio-renal-metabolic vulnerability outcomes.

Outcome	Active vs. inactive, PR (95% CI)
High CRM vulnerability	1.121 (1.051–1.197)
Severe CRM vulnerability	1.267 (1.067–1.504)
Subclinical high CRM vulnerability	1.109 (1.025–1.199)
High CRM vulnerability using the stricter renal definition	1.101 (1.016–1.193)

Results are from fully adjusted multiply imputed modified Poisson regression models with robust standard errors. Models were adjusted for age, sex, current smoking, current drinking, and BMI. Inactive participants were used as the reference group. The active group combined participants with low and moderate-to-high routine-recorded physical activity. PR, prevalence ratio; CI, confidence interval; CRM, cardio-renal-metabolic; BMI, body mass index. The original three-category exposure analysis is provided as a secondary sensitivity analysis in Supplementary Table S5.

Because the low recorded-activity subgroup was small, the inactive-versus-active contrast was used as the revised primary exposure comparison. The original three-category exposure variable was retained only as a secondary sensitivity analysis to preserve the structure of the routine record. These secondary results showed that the positive direction was mainly observed in the moderate-to-high recorded-activity category, whereas estimates for the low recorded-activity subgroup were imprecise and should be interpreted cautiously (Supplementary Table S5). Therefore, the primary interpretation of the study is based on the binary routine-recorded physical activity contrast rather than on the small low recorded-activity category.

### 3.3 Domain-specific associations

**Figure 3:**
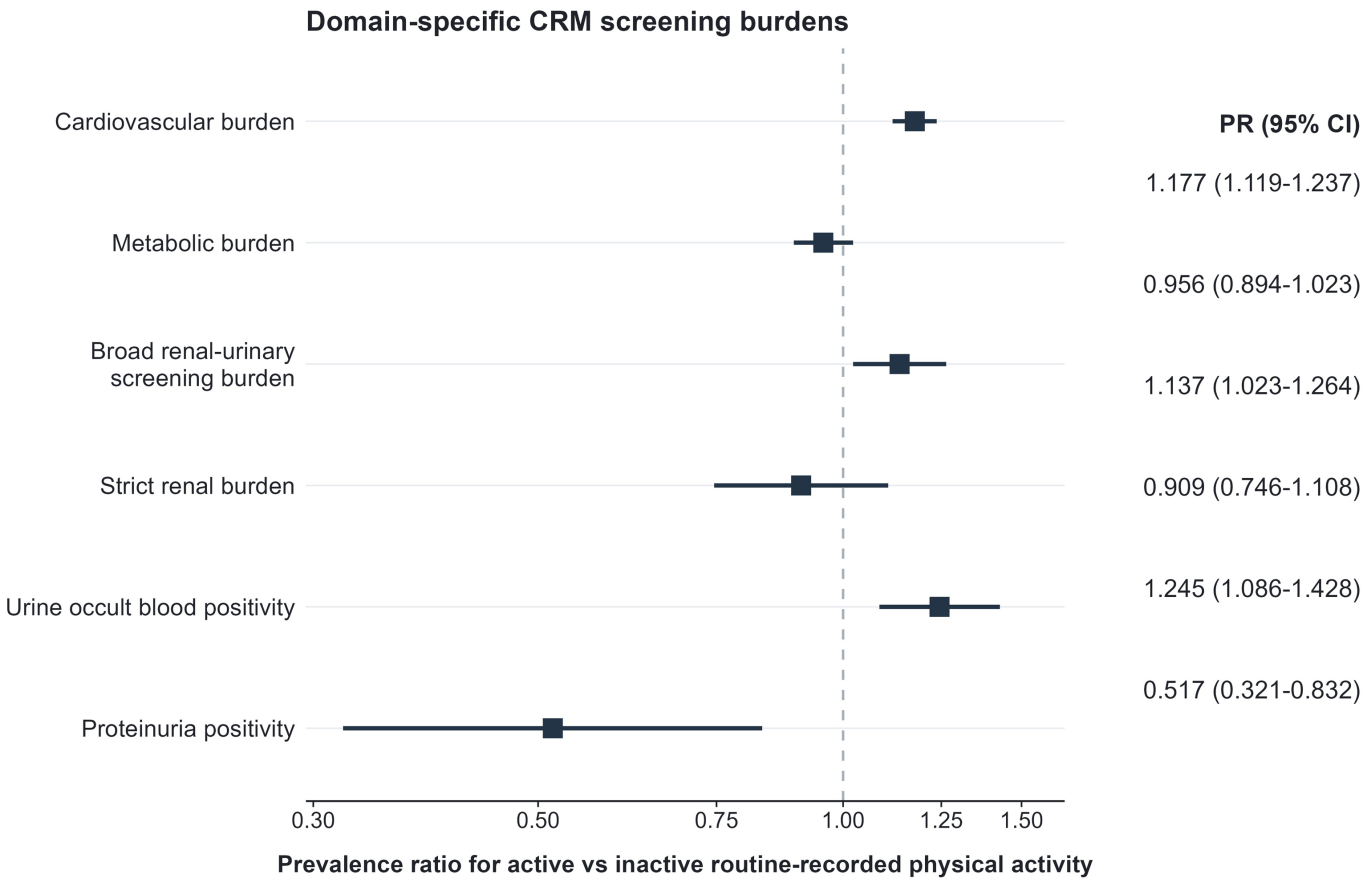
Domain-specific associations of routine-recorded physical activity with cardiovascular, metabolic, and renal–urinary screening burdens. Forest plot showing fully adjusted prevalence ratios and 95% confidence intervals for active versus inactive routine-recorded physical activity across domain-specific cardio-renal-metabolic screening burdens. Broad renal–urinary screening burden included urine occult blood positivity, proteinuria positivity, and/or reduced eGFR. The stricter renal screening burden was defined by reduced eGFR and/or proteinuria positivity and was interpreted as a screening signal rather than as a diagnosis of chronic kidney disease. Models were estimated using multiply imputed data and modified Poisson regression with robust standard errors, adjusted for age, sex, current smoking, current drinking, and BMI. Inactive participants were used as the reference group. CRM, cardio-renal-metabolic; CI, confidence interval; eGFR, estimated glomerular filtration rate; PR, prevalence ratio.

Domain-specific analyses based on the primary binary exposure contrast are presented in Table 3 and Figure 3. In the fully adjusted multiply imputed models, active routine-recorded physical activity was associated with a higher prevalence of cardiovascular burden (PR = 1.177, 95% CI: 1.119–1.237) and broad renal–urinary screening burden (PR = 1.137, 95% CI: 1.023–1.264) compared with inactivity. In contrast, no comparable positive association was observed for metabolic burden (PR = 0.956, 95% CI: 0.894–1.023) or the stricter renal screening burden defined by reduced eGFR and/or proteinuria positivity (PR = 0.909, 95% CI: 0.746–1.108).

**Table 3:** Domain-specific binary associations between routine-recorded physical activity and cardiovascular, metabolic, and renal–urinary screening burdens.

Outcome	Active vs. inactive, PR (95% CI)	Analysis
Cardiovascular burden	1.177 (1.119–1.237)	MI
Metabolic burden	0.956 (0.894–1.023)	MI
Broad renal–urinary screening burden	1.137 (1.023–1.264)	MI
Stricter renal screening burden	0.909 (0.746–1.108)	MI
Urine occult blood positivity	1.245 (1.086–1.428)	MI
Proteinuria positivity	0.517 (0.321–0.832)	MI

Results are from fully adjusted multiply imputed modified Poisson regression models with robust standard errors. Models were adjusted for age, sex, current smoking, current drinking, and BMI. Inactive participants were used as the reference group. The active group combined participants with low and moderate-to-high routine-recorded physical activity. Cardiovascular burden was defined by elevated blood pressure, hypertension history, or antihypertensive treatment. Metabolic burden was defined by hyperglycaemia, diabetes history, glucose-lowering treatment, hypertriglyceridaemia, low HDL-C, or lipid-lowering treatment. Broad renal–urinary screening burden was defined by reduced eGFR, proteinuria positivity, or urine occult blood positivity. Stricter renal screening burden was defined by reduced eGFR and/or proteinuria positivity and was interpreted as a screening signal rather than as a diagnosis of chronic kidney disease. PR, prevalence ratio; CI, confidence interval; MI, multiply imputed analysis; BMI, body mass index; HDL-C, high-density lipoprotein cholesterol; eGFR, estimated glomerular filtration rate.

Further component-level decomposition indicated that the broad renal–urinary screening signal was observed mainly in relation to urine occult blood positivity (PR = 1.245, 95% CI: 1.086–1.428), whereas proteinuria positivity showed an inverse association (PR = 0.517, 95% CI: 0.321–0.832). Because UACR was unavailable and the stricter renal screening burden did not show a parallel positive pattern, these renal–urinary findings should be interpreted as broad screening signals rather than as evidence of established kidney disease.

### 3.4 Supplementary robustness analyses

Supplementary robustness analyses further supported the stability and interpretability of the revised primary analysis. First, the primary binary exposure analyses showed positive associations between active routine-recorded physical activity and the main and alternative CRM vulnerability outcomes in the fully adjusted multiply imputed models (Supplementary Table S4). Second, the original three-category exposure analysis was retained as a secondary sensitivity analysis; these results showed a consistent positive direction for the moderate-to-high recorded activity group, whereas the low recorded-activity subgroup yielded imprecise estimates because of its small sample size (Supplementary Table S5). Third, binary subgroup analyses showed that the positive direction of association was broadly preserved across sex, age, and BMI strata, although estimates were less precise in some strata and should be interpreted exploratorily (Supplementary Table S6). Fourth, analyses excluding participants with diabetes showed a similar positive direction for the moderate-to-high recorded activity group in the secondary three-category framework, suggesting that the observed pattern was not solely explained by participants with established diabetes (Supplementary Table S7). Finally, formal interaction analyses did not provide strong evidence that the association varied consistently by sex, age group, or BMI (Supplementary Table S8). These findings support the robustness of the main association whilst reinforcing that subgroup, interaction, and secondary three-category results should be interpreted cautiously.

## 4 Discussion

### 4.1 Principal findings

In this county-based sample of older adults from Wangkui County, Heilongjiang Province, active routine-recorded physical activity was associated with a higher prevalence of screening-defined cardio-renal-metabolic (CRM) vulnerability compared with inactivity, as shown in the primary analysis. This pattern remained directionally consistent across alternative outcome definitions, including severe vulnerability, subclinical vulnerability, and the stricter renal version of the composite outcome. Domain-specific analyses showed that the overall association was observed mainly for cardiovascular burden and broader renal–urinary screening signals, whereas no comparable positive pattern was observed for the metabolic domain and the stricter renal indicators did not show parallel elevation. Taken together, these findings indicate that, within this routine screening dataset, being recorded as active co-occurred with a less favourable multisystem screening profile in this rural ageing population, although the pattern was selective rather than generalised across all domains.

### 4.2 Relationship to previous publication and scope of the current analysis

These findings should be interpreted transparently in relation to our previous publication from the same Wangkui County health-examination platform (22). The two studies were derived from the same source population; therefore, the present analysis should be understood as a transparent secondary analysis of the same routine screening platform rather than as evidence from an independent cohort. The earlier article examined hypertension as a disease-specific cardiovascular outcome and evaluated blood-pressure-free obesity–metabolic phenotypes, a quantitative physical-activity volume index based on recorded frequency and duration, and leakage-free prediction models. In contrast, the present study uses the routine activity record as a coarse inactive-versus-active screening indicator rather than as a validated measure of physical activity, exercise dose, energy expenditure, intensity, or activity domain.

The present study extends that framework conceptually and analytically by using a screening-defined multidomain CRM vulnerability outcome rather than hypertension alone. This CRM outcome should be interpreted as an operational screening construct reflecting the co-occurrence of routine examination abnormalities, not as a clinically validated disease phenotype or diagnostic cardio-renal-metabolic syndrome. Although the cardiovascular domain in the current composite necessarily includes blood-pressure and hypertension-related information, the primary endpoint required involvement of at least two CRM domains and was accompanied by alternative outcome definitions, stricter renal sensitivity analyses, and domain-level decomposition. Accordingly, the cardiovascular component should be viewed as one element within the composite screening framework, not as a new standalone hypertension analysis.

The progression of evidence is therefore conceptual and analytic rather than cohort-based: the analysis moves from disease-specific hypertension modelling to a broader routine-screening vulnerability framework that examines how cardiovascular, metabolic, and renal–urinary screening signals co-occur in relation to routine-recorded activity status. Because the study is cross-sectional, these findings indicate associations and co-occurrence patterns only; they should not be interpreted as causal evidence that routine-recorded activity increases CRM vulnerability or that structured physical activity is harmful. Accordingly, the current findings should not be interpreted as an independent replication of the prior hypertension result, but as a transparent multidomain extension of the same county-level screening platform with a different exposure operationalisation, endpoint hierarchy, statistical framing, and interpretive purpose.

### 4.3 Ageing, context, and the meaning of routine-recorded activity in a cold-climate agricultural county

The present findings should be interpreted within the specific social and environmental context of Wangkui County rather than through the conventional assumption that greater physical activity is uniformly health promoting. In many public health and exercise-based studies, higher levels of activity are associated with better cardiometabolic health and healthier ageing trajectories (5, 14). However, the activity item used in the present study was derived from a routine county health-examination record rather than from a validated physical activity questionnaire. It did not distinguish leisure-time exercise, occupational or agricultural labour, domestic activity, transport-related movement, activity intensity, activity duration, or total energy expenditure. Therefore, the exposure should be interpreted as routine-recorded physical activity rather than as a direct measure of exercise dose or labour burden (14, 15, 26, 27).

This contextual distinction is central to interpreting the current results. In the present dataset, recorded activity should be understood as a coarse routine-recorded activity indicator rather than as a direct measure of leisure-time exercise, occupational or agricultural labour, domestic activity, transport-related movement, or total activity dose. For older adults in Wangkui County, being recorded as active may reflect heterogeneous daily movement, including household responsibilities, agricultural routines, transport-related activity, livelihood-related demands, and discretionary exercise, but the routine record itself cannot distinguish these sources. Under such conditions, an active routine record should not automatically be interpreted as structured health-promoting exercise. It may also coexist with cumulative workload, limited recovery, chronic disease burden, and constrained access to preventive health resources. From this perspective, the present study does not challenge the general value of physical activity. Rather, it suggests that the health meaning of movement in later life depends on its social function, workload characteristics, and recovery conditions. This distinction is especially relevant at the intersections of ageing, activity context, and health, where routine-recorded activity may capture mixed daily movement rather than behavioural vitality alone (9, 21, 28).

### 4.4 Interpretation of the domain-specific vulnerability pattern

An important strength of this study is that the main association was examined not only through a composite outcome but also through domain-specific decomposition (1, 3, 29). This approach helped refine the interpretation of the composite endpoint and reduced the risk of overinterpreting CRM vulnerability as a uniform multisystem condition. Active routine-recorded physical activity was associated with cardiovascular burden and broad renal–urinary screening burden, whereas metabolic burden did not show a comparable positive association. Thus, the observed association should be interpreted as a domain-selective screening pattern rather than as evidence of generalised cardio-renal-metabolic abnormality. This pattern suggests that the observed relationship was not a uniform multisystem abnormality pattern, but rather a more selective screening-vulnerability profile.

The renal findings require particularly cautious interpretation. Active routine-recorded physical activity was associated with the broader renal–urinary screening domain and with urine occult blood positivity, but not with the stricter renal screening burden defined by reduced eGFR or proteinuria. In addition, proteinuria showed an inverse association. Importantly, UACR was not available in the routine health-examination records, and urine occult blood positivity is a non-specific screening marker that may reflect heterogeneous urinary findings. These results therefore should not be interpreted as indicating established kidney disease, clinically adjudicated chronic kidney disease, or renal abnormalities attributable to activity. A more defensible interpretation is that the renal–urinary portion of the composite vulnerability pattern was observed primarily through broader screening signals, particularly urine occult blood positivity, rather than through consistent evidence from stricter renal indicators. Given the cross-sectional design, these findings should not be interpreted causally.

This domain-specific divergence is important for interpretation. Without separating broader and stricter renal indicators, the overall composite result could easily have been overinterpreted. Instead, the current findings support a more restrained conclusion: the observed association appears to reflect a specific configuration of cardiovascular burden and broader renal–urinary screening abnormalities rather than as evidence of a generalised cardio-renal-metabolic abnormality pattern (3). Because the cardiovascular domain partly included blood-pressure- and hypertension-related indicators, and because the positive renal–urinary pattern was observed mainly for a broad urine-screening marker, the composite outcome should be interpreted as an operational screening construct rather than as a clinically validated disease phenotype. In this context, the broader renal–urinary screening signal should not be treated as synonymous with established kidney damage. Rather, it may serve as a pragmatic signal for follow-up prioritisation within routine county-level screening, whilst requiring confirmation by more specific renal measures such as UACR, repeated urinalysis, and clinically adjudicated kidney-function assessment in future studies.

### 4.5 Public health implications

These findings have practical implications for public health strategies targeting older adults in rural agricultural settings, provided that they are interpreted as screening-level associations rather than causal evidence or clinical risk predictions. First, they suggest that older adults who are recorded as active in routine health-examination data should not automatically be assigned lower screening or follow-up priority. In county-level screening practise, recorded activity may coexist with, rather than exclude, multisystem vulnerability. This is particularly relevant in settings such as Wangkui County, where older adults may appear active because of mixed daily movement, household responsibilities, agricultural routines, transport-related activity, livelihood demands, or discretionary exercise, without the routine record distinguishing these sources. Therefore, routine-recorded activity should be treated as a contextual screening indicator, not as a validated measure of exercise dose, activity intensity, energy expenditure, or health-promoting physical activity.

Second, the results support a more differentiated approach to activity interpretation and health education in later life. Public health recommendations often emphasise increasing physical activity to prevent chronic disease and support healthy ageing. However, for older adults in rural agricultural environments, simply encouraging more activity without considering workload structure, recovery, seasonal constraints, and existing screening vulnerability may be insufficient or conceptually misleading. The more useful distinction may be between structured health-promoting activity and heterogeneous routine-recorded daily movement. This does not challenge the established value of structured physical activity or leisure-time exercise; rather, it suggests that activity records in later life should be interpreted in relation to work context, bodily recovery, environmental exposure, and structural conditions. Accordingly, county-level health workers should avoid assuming that “being active” in routine records necessarily indicates adequate protective exercise or lower screening priority.

Third, the present findings highlight the potential public health utility of county-level screening frameworks that move beyond single-disease identification. In this study, the screening-defined CRM vulnerability framework captured a broader screening pattern that may help inform follow-up prioritisation than any one disease outcome alone. However, this framework should be understood as an operational screening construct rather than as a clinically validated disease phenotype or diagnostic cardio-renal-metabolic syndrome. For ageing rural populations, especially those in cold-climate agricultural regions, such multisystem screening approaches may be useful for screening interpretation, follow-up prioritisation, resource allocation, and hypothesis generation, rather than for establishing diagnosis, causal inference, or clinical classification (1–3, 5, 30, 31). In practical terms, the findings suggest that routine health-surveillance data may help identify older adults who warrant more careful blood-pressure review, metabolic assessment, renal–urinary follow-up, and lifestyle-context evaluation, whilst more specific clinical assessments remain necessary before any disease-level interpretation is made.

### 4.6 Strengths and limitations

This study has several strengths. It used real-world county-level screening data from a rural older population in northeastern China, a setting that remains underrepresented in the literature. It also moved beyond single-disease analysis by constructing a screening-defined cardio-renal-metabolic (CRM) vulnerability framework and examining its consistency across alternative outcome definitions. Importantly, this framework was used as an operational screening construct rather than as a clinically validated disease phenotype, which allowed the analysis to focus on routine examination patterns and follow-up prioritisation. In response to the small low-activity subgroup, the revised analysis used the binary inactive-versus-active comparison as the primary exposure contrast, whilst retaining the original three-category exposure only as a secondary sensitivity analysis. In addition, the analysis incorporated multiple robustness cheques, including multiply imputed primary models, complete-case comparisons, subgroup analyses, exclusion of participants with diabetes, interaction analyses, and domain-specific decomposition. The domain-specific decomposition was particularly important because it clarified that the composite association was not uniformly present across all CRM domains. Importantly, the relationship between the present analysis and our previous hypertension-focused publication from the same county-level screening platform has been transparently stated in the Introduction (22), Methods, Discussion, and Acknowledgements. Accordingly, the current manuscript should be interpreted as a multidomain screening-vulnerability analysis rather than as a disease-specific hypertension analysis or independent evidence from a separate cohort. Taken together, these features enhance the transparency, interpretive caution, and internal consistency of the observed screening-defined association pattern.

Several limitations should also be acknowledged. First, the cross-sectional design precludes establishing temporal order between routine-recorded physical activity and the observed vulnerability profile; reverse causation and bidirectional relationships therefore cannot be excluded. All findings should therefore be interpreted as associations and co-occurrence patterns, not as causal effects. Second, the activity measure was relatively coarse and was collected from a routine health-examination item rather than from a validated physical activity questionnaire. It did not capture activity intensity, duration, energy expenditure, occupational workload, leisure-time exercise, domestic activity, transport-related activity, seasonal variation, or recovery conditions. Therefore, the exposure should be interpreted as routine-recorded physical activity status from a health-examination record, rather than as a precise measure of exercise dose, total physical activity, structured exercise, or labour burden. Third, the original low-activity subgroup was small, which limited precision for three-category comparisons. This was the reason for defining inactive versus active routine-recorded physical activity as the revised primary exposure contrast. The current findings should therefore not be interpreted as evidence against leisure-time exercise or structured physical activity.

Fourth, the screening-defined CRM vulnerability outcome was an operational construct based on routine examination information. It was not designed to diagnose a clinically validated cardio-renal-metabolic disease phenotype, establish prognosis, or replace disease-specific clinical assessment. Fifth, the renal–urinary findings require conservative interpretation. The broader renal–urinary signal was observed mainly in relation to urine occult blood positivity, whereas the stricter renal domain based on reduced eGFR and/or proteinuria did not show a parallel positive pattern. In addition, UACR was not available in the routine health-examination records, and urine occult blood positivity is a non-specific screening marker. Accordingly, the renal–urinary component should be interpreted as a broader screening signal rather than as direct evidence of stable renal injury, clinically adjudicated chronic kidney disease, or kidney damage attributable to activity itself.

Sixth, residual confounding remains possible despite multivariable adjustment and multiple imputation. Unmeasured factors such as work intensity, seasonality of labour, occupational history, functional status, household support, diet, medication use, healthcare access, socioeconomic conditions, and recovery opportunity may have been related to both recorded activity patterns and screening-defined health outcomes. Seventh, the present study used the same 2025 Wangkui County routine health-examination source platform as a previous publication by our author group (22). Although the current manuscript addressed a distinct research question, used a different primary exposure operationalisation, constructed a multidomain CRM vulnerability outcome, and applied a different statistical framework, the source population substantially overlapped with that of the previous hypertension-focused article. Therefore, the current findings should be interpreted as a transparent secondary analysis and multidomain extension from the same routine screening platform, not as evidence from an independent cohort. In addition, because the cardiovascular domain included blood-pressure and hypertension-related indicators, the domain-specific cardiovascular findings should be understood as contributors to the composite CRM vulnerability construct rather than as a new disease-specific hypertension analysis. Finally, the study population was drawn from a county-level screening context in Wangkui, and the findings are therefore most directly applicable to older adults living in cold-climate agricultural rural settings rather than to urban populations or cohorts characterised primarily by leisure-time exercise (32). External validation in other rural and urban populations, preferably using prospective designs and refined physical-activity and renal measurements, will be necessary before broader generalisation or clinical interpretation.

### 4.7 Future directions

Future research should distinguish routine-recorded physical activity from structured exercise, occupational or agricultural labour, domestic tasks, transport-related movement, and total energy expenditure using more refined exposure assessment, prospective designs, and comparative evidence from other rural ageing populations (14, 15, 26). Longitudinal studies with better characterisation of work intensity, seasonal activity patterns, recovery conditions, functional status, and healthcare access will be particularly important for clarifying whether the observed association reflects cumulative workload exposure, reverse causation, selective survival, screening capture, or a combination of these possible explanations. Such work will be essential for improving how routine activity records are interpreted at the intersections of ageing, activity context, and health in rural settings (15, 26, 31).

## 5 Conclusion

Amongst older adults in Wangkui County, Heilongjiang Province, active routine-recorded physical activity status co-occurred with a higher prevalence of screening-defined CRM vulnerability compared with inactivity. This association should be interpreted as a screening-context signal rather than as evidence of a harmful causal effect of physical activity. The exposure was derived from a routine health-examination item and did not distinguish leisure-time exercise, occupational or agricultural activity, domestic work, transport-related movement, seasonal workload, intensity, duration, total energy expenditure, or exercise dose. The CRM outcome was an operational screening construct rather than a clinically validated disease phenotype. The observed pattern was concentrated mainly in cardiovascular indicators and broad renal–urinary screening signals, whilst stricter renal indicators did not show a consistent parallel pattern. Therefore, the renal–urinary findings should be interpreted conservatively, especially because UACR was unavailable and the broader renal–urinary signal was observed mainly in relation to urine occult blood positivity. These findings do not challenge the established health benefits of structured physical activity or leisure-time exercise. Instead, they suggest that routine activity records in cold-climate agricultural older populations may capture heterogeneous daily movement that can coexist with multisystem screening vulnerability. Future prospective studies using refined activity assessment are needed to clarify temporality, activity domains, workload context, recovery conditions, and clinical significance. From an ageing and public health perspective, these results highlight the need to interpret routine activity records in older rural adults as contextual screening information that may guide follow-up prioritisation, rather than as a substitute for validated physical-activity assessment or clinical disease classification.

## Data Availability

The data analyzed in this study are not publicly available because they were generated from a government-organised annual health-examination programme and were released to the research team under a data-use agreement with the local Health Bureau. The authors are not permitted to publicly deposit or transfer the raw individual-level data. De-identified data necessary to reproduce the reported analyses may be made available by the corresponding author on reasonable request, subject to approval by the data custodian/local Health Bureau and applicable institutional, legal, and ethical requirements.
