# Supplementary material for: Routine-recorded physical activity and screening-defined cardio-renal-metabolic vulnerability amongst older adults in a cold-climate agricultural county in Northeast China
Source: Front Public Health. 2026 Jul 7;14:1831345. doi: 10.3389/fpubh.2026.1831345 (PMC13386333; doi:10.3389/fpubh.2026.1831345)
Supplement: Supplementary file 1 [file Table_1.DOCX]

**Supplementary Table S1. Source physical-activity item, exported raw values, and analytic recoding rules**

| **Source item** | **English translation** | **Exported raw value** | **Analytic interpretation** | **Three-category classification** | **Primary binary classification** | **n** | **% of total sample** |
| --- | --- | --- | --- | --- | --- | --- | --- |
| Physical-exercise frequency item | Frequency of physical exercise | 0 | No recorded physical-exercise frequency | Inactive | Inactive | 852 | 37.53 |
| Physical-exercise frequency item | Frequency of physical exercise | 1 | Low recorded physical activity | Low recorded activity | Active | 46 | 2.03 |
| Physical-exercise frequency item | Frequency of physical exercise | 3 | Moderate-to-high recorded physical activity | Moderate-to-high recorded activity | Active | 1317 | 58.02 |
| Physical-exercise frequency item | Frequency of physical exercise | Missing | Missing frequency record | Missing | Missing | 55 | 2.42 |

**Note.** The exposure was derived from the physical-exercise frequency field in the lifestyle section of the county health-examination form. The field label was translated from Chinese as “frequency of physical exercise” (“体育锻炼频率”). The exported analytic dataset contained raw values of 0, 1, and 3, as well as missing records; no raw value of 2 was observed. The binary inactive-versus-active classification was used as the revised primary exposure because the low recorded-activity subgroup was small. The original three-category classification was retained as a secondary analysis. Percentages were calculated using the total analytic sample as the denominator.

**Supplementary Table S2. Availability of companion exercise-related fields according to exported physical-exercise frequency values**

| **Exported raw value for physical-exercise frequency** | **n** | **Non-missing exercise duration per session** | **Non-missing years of continued exercise** | **Non-missing exercise type** | **Median exercise duration, min [IQR]** | **Median years of continued exercise [IQR]** |
| --- | --- | --- | --- | --- | --- | --- |
| 0 | 852 | 0 / 852 (0.00%) | 0 / 852 (0.00%) | 0 / 852 (0.00%) | — | — |
| 1 | 46 | 43 / 46 (93.48%) | 42 / 46 (91.30%) | 40 / 46 (86.96%) | 60 [30, 60] | 5 [2, 10] |
| 3 | 1317 | 1312 / 1317 (99.62%) | 1295 / 1317 (98.33%) | 1296 / 1317 (98.41%) | 60 [60, 60] | 7 [4, 10] |
| Missing | 55 | 11 / 55 (20.00%) | 11 / 55 (20.00%) | 11 / 55 (20.00%) | 60 [40, 120] | 6 [2, 11.5] |

**Note.** Companion exercise-related fields included exercise duration per session, years of continued exercise, and exercise type. These fields were used only to document the structure and internal consistency of the routine physical-activity record. They were not used to construct the primary exposure because the study aimed to evaluate the screening meaning of the routinely recorded physical-exercise frequency item rather than to estimate total physical activity dose.

**Supplementary Table S3. Operational definitions of exposure, domain-specific screening burdens, and composite vulnerability outcomes**

| **Variable / outcome** | **Operational definition** |
| --- | --- |
| Routine-recorded physical activity group | Classified into inactive, low recorded activity, and moderate-to-high recorded activity according to the study-specific recoding of the exported physical-exercise frequency field from the routine health-examination record. This variable was interpreted as a routine-recorded activity indicator rather than as a validated measure of physical activity dose, intensity, energy expenditure, or activity domain. |
| Binary routine-recorded physical activity | Inactive vs. active, where active combined the low recorded activity and moderate-to-high recorded activity groups. This binary contrast was used as the primary exposure because the low recorded-activity subgroup was small. |
| Cardiovascular burden | SBP ≥140 mmHg, DBP ≥90 mmHg, hypertension history, or antihypertensive treatment. |
| Metabolic burden | FPG ≥7.0 mmol/L, diabetes history, glucose-lowering treatment, TG ≥1.7 mmol/L, low HDL-C, or lipid-lowering treatment. |
| Broad renal–urinary screening burden | eGFR <60 mL/min/1.73 m², proteinuria positivity, or urine occult blood positivity. This was interpreted as a broad renal–urinary screening signal rather than as a clinically adjudicated kidney disease definition. |
| Strict renal burden | eGFR <60 mL/min/1.73 m² or proteinuria positivity. |
| High CRM vulnerability | Involvement of at least two of the three prespecified domains: cardiovascular burden, metabolic burden, and broad renal–urinary screening burden. |
| Severe CRM vulnerability | Concurrent involvement of all three prespecified domains. |
| Subclinical high CRM vulnerability | Involvement of at least two domains based on objective screening abnormalities only, without relying on prior disease history or treatment status. |
| High CRM vulnerability using stricter renal definition | Involvement of at least two domains when the renal component was defined using reduced eGFR or proteinuria only, instead of the broad renal–urinary screening burden. |

**Note.** CRM, cardio-renal-metabolic; SBP, systolic blood pressure; DBP, diastolic blood pressure; FPG, fasting plasma glucose; TG, triglycerides; HDL-C, high-density lipoprotein cholesterol; eGFR, estimated glomerular filtration rate. The CRM outcomes were screening-based operational constructs designed to describe co-occurrence patterns in routine health-examination data. They were not clinically validated disease phenotypes, diagnostic cardio-renal-metabolic syndromes, or prognostic classifications. UACR was not available in the routine health-examination records; therefore, the renal–urinary domain, especially the broad renal–urinary screening burden that included urine occult blood positivity, should be interpreted as a screening signal rather than as evidence of established chronic kidney disease or stable renal injury.

**Supplementary Table S4. Primary binary analyses for the associations between routine-recorded physical activity and screening-defined cardio-renal-metabolic vulnerability outcomes**

| **Outcome** | **Active vs inactive, PR (95% CI)** | **Analysis** |
| --- | --- | --- |
| High CRM vulnerability | 1.121 (1.051–1.197) | MI |
| Severe CRM vulnerability | 1.267 (1.067–1.504) | MI |
| Subclinical high CRM vulnerability | 1.109 (1.025–1.199) | MI |
| High CRM vulnerability using the stricter renal definition | 1.101 (1.016–1.193) | MI |

**Note.** The binary exposure was used as the revised primary exposure contrast because the low recorded-activity subgroup was small. Participants with low and moderate-to-high routine-recorded physical activity were combined into the active group, and inactive participants were used as the reference group. PR indicates prevalence ratio; CI, confidence interval; CRM, cardio-renal-metabolic; MI, multiply imputed analysis. Results are from fully adjusted modified Poisson regression models with robust standard errors, adjusted for age, sex, current smoking, current drinking, and BMI.

**Supplementary Table S5. Secondary three-category analyses for the associations between routine-recorded physical activity and screening-defined cardio-renal-metabolic vulnerability outcomes**

| **Outcome** | **Model** | **Low activity vs inactive, PR (95% CI)** | **Moderate-to-high activity vs inactive, PR (95% CI)** | **Analysis** |
| --- | --- | --- | --- | --- |
| High CRM vulnerability | Model 0 | 1.193 (0.984–1.448) | 1.167 (1.093–1.245) | MI |
| High CRM vulnerability | Model 1 | 1.169 (0.962–1.421) | 1.153 (1.079–1.231) | MI |
| High CRM vulnerability | Model 2 | 1.146 (0.945–1.390) | 1.120 (1.049–1.196) | MI |
| Severe CRM vulnerability | Model 0 | 0.944 (0.488–1.826) | 1.328 (1.123–1.570) | MI |
| Severe CRM vulnerability | Model 1 | 0.915 (0.471–1.778) | 1.311 (1.106–1.552) | MI |
| Severe CRM vulnerability | Model 2 | 0.901 (0.463–1.752) | 1.280 (1.078–1.520) | MI |
| Subclinical high CRM vulnerability | Model 0 | 0.924 (0.691–1.236) | 1.166 (1.084–1.253) | MI |
| Subclinical high CRM vulnerability | Model 1 | 0.921 (0.687–1.233) | 1.148 (1.065–1.238) | MI |
| Subclinical high CRM vulnerability | Model 2 | 0.916 (0.683–1.228) | 1.115 (1.031–1.207) | MI |
| High CRM vulnerability using stricter renal definition | Model 0 | 1.140 (0.893–1.456) | 1.106 (1.022–1.197) | MI |
| High CRM vulnerability using stricter renal definition | Model 1 | 1.120 (0.876–1.432) | 1.111 (1.025–1.204) | MI |
| High CRM vulnerability using stricter renal definition | Model 2 | 1.108 (0.864–1.421) | 1.100 (1.015–1.193) | MI |

**Note.** This table retains the original three-category routine-recorded physical-activity classification as a secondary analysis. Inactive participants were used as the reference group. Model 0 was unadjusted. Model 1 was adjusted for age, sex, current smoking, and current drinking. Model 2 was additionally adjusted for BMI. PR indicates prevalence ratio; CI, confidence interval; CRM, cardio-renal-metabolic; MI, multiply imputed analysis. Because the low recorded-activity subgroup was small, estimates for this subgroup should be interpreted cautiously; the primary exposure contrast in the revised analysis was the binary inactive-versus-active comparison shown in Supplementary Table S4.

**Supplementary Table S6. Binary subgroup analyses for the association between routine-recorded physical activity and high screening-defined cardio-renal-metabolic vulnerability**

| **Stratum** | **N** | **Active vs inactive, PR (95% CI)** | **Analysis** |
| --- | --- | --- | --- |
| Male | 1,081 | 1.172 (1.048–1.309) | MI |
| Female | 1,134 | 1.083 (1.001–1.171) | MI |
| Age <75 years | 1,484 | 1.132 (1.044–1.227) | MI |
| Age ≥75 years | 731 | 1.102 (0.984–1.233) | MI |
| BMI <24 kg/m² | 1,040 | 1.160 (1.042–1.292) | MI |
| BMI ≥24 kg/m² | 1,173 | 1.105 (1.018–1.200) | MI |

**Note.** Results are from fully adjusted multiply imputed modified Poisson regression models with robust standard errors. The outcome was high screening-defined cardio-renal-metabolic vulnerability. Inactive participants were used as the reference group. The active group combined participants with low and moderate-to-high routine-recorded physical activity. Sex-stratified models were adjusted for age, current smoking, current drinking, and BMI. Age-stratified models were adjusted for sex, current smoking, current drinking, and BMI. BMI-stratified models were adjusted for age, sex, current smoking, and current drinking. N denotes the number of observed participants with non-missing physical activity classification and the corresponding stratification variable. PR, prevalence ratio; CI, confidence interval; MI, multiply imputed analysis; BMI, body mass index.

**Supplementary Table S7. Secondary three-category sensitivity analysis excluding participants with diabetes**

| **Model** | **Low activity vs inactive, PR (95% CI)** | **Moderate-to-high activity vs inactive, PR (95% CI)** | **N** |
| --- | --- | --- | --- |
| Model 0 | 1.225 (0.998–1.504) | 1.144 (1.063–1.232) | 1,941 |
| Model 1 | 1.200 (0.974–1.479) | 1.126 (1.044–1.214) | 1,941 |
| Model 2 | 1.177 (0.957–1.447) | 1.088 (1.010–1.173) | 1,941 |

**Note.** This sensitivity analysis was restricted to participants without diabetes. Model 0 was unadjusted. Model 1 was adjusted for age, sex, current smoking, and current drinking. Model 2 was additionally adjusted for BMI. Inactive participants were used as the reference group. These analyses retained the original three-category exposure for secondary exploration; the revised primary exposure contrast was the binary inactive-versus-active comparison.

**Supplementary Table S8. Interaction analyses for binary routine-recorded physical activity with sex, age group, and BMI**

| **Interaction model** | **Term** | **PR (95% CI)** | **Analysis** |
| --- | --- | --- | --- |
| PA × sex | Active vs inactive among females | 1.076 (0.996–1.163) | MI |
| PA × sex | Active × male | 1.099 (0.963–1.255) | MI |
| PA × age group | Active vs inactive among age <75 years | 1.131 (1.044–1.226) | MI |
| PA × age group | Active × age ≥75 years | 0.974 (0.850–1.116) | MI |
| PA × BMI | Active vs inactive at BMI = 24 kg/m² | 1.122 (1.048–1.202) | MI |
| PA × BMI | Active × BMI, per 1 kg/m² | 0.999 (0.982–1.016) | MI |

**Note.** Results are from fully adjusted multiply imputed modified Poisson regression models with robust standard errors. The outcome was high screening-defined cardio-renal-metabolic vulnerability. Inactive participants were used as the reference group. The active group combined participants with low and moderate-to-high routine-recorded physical activity. For the PA × sex model, females were the reference sex group. For the PA × age group model, participants aged <75 years were the reference age group. For the PA × BMI model, BMI was modelled as a continuous modifier centred at 24 kg/m². Interaction terms represent multiplicative interaction prevalence ratios. Models were adjusted for age, sex, current smoking, current drinking, and BMI as appropriate; the stratification or interaction variable was not redundantly adjusted where it was already included as the effect modifier. PR, prevalence ratio; CI, confidence interval; MI, multiply imputed analysis; PA, physical activity; BMI, body mass index.
